# Do Individuals with Spinal Cord Injury Benefit from Semi-Immersive Virtual Reality Cognitive Training? Preliminary Results from an Exploratory Study on an Underestimated Problem

**DOI:** 10.3390/brainsci13060945

**Published:** 2023-06-13

**Authors:** Maria Grazia Maggio, Mirjam Bonanno, Alfredo Manuli, Maria Pia Onesta, Rosaria De Luca, Angelo Quartarone, Rocco Salvatore Calabrò

**Affiliations:** 1 Department of Biomedical and Biotechnological Science, University of Catania, Via S. Sofia 64, 95125 Catania, Italy; mariagraziamay@gmail.com; 2 IRCCS Centro Neurolesi “Bonino Pulejo”, S. S. 113, Contrada Casazza, 98124 Messina, Italy; mirjam.bonanno@irccsme.it (M.B.); rosaria.deluca@irccsme.it (R.D.L.); angelo.quartarone@irccsme.it (A.Q.); 3 Rehabilitation Unit, AOU Policlinico “G. Martino”, 98124 Messina, Italy; manulialfredo@gmail.com; 4 Spinal Cord Unit, Cannizzaro Hospital, 95126 Catania, Italy; mp.onesta@gmail.com

**Keywords:** spinal cord injury, neurorehabilitation, semi-immersive virtual reality, cognitive training

## Abstract

A spinal cord injury (SCI) is damage to any part of the spinal cord, caused by traumatic or non-traumatic events. Clinically, SCI is associated with partial or complete loss of motor, sensory, and autonomic functions below the site of injury. However, cognitive alterations in specific domains can also occur. The aim of this study was to evaluate the effects of semi-immersive virtual reality (VR) cognitive training (using the BTS Nirvana, Italy) in promoting global functional recovery in patients with SCI. Forty-two SCI patients were included in this retrospective case-control study, and the analysis was carried out using an electronic data retrieval system. The enrolled patients were divided into two groups with the same demographic and medical characteristics: the control group (CG: 21 patients) participated in traditional therapy, whereas the experimental group (EG: 21 patients) received training using semi-immersive VR. In both groups, there were patients with A- or B-grade impairments according to the American Spinal Injury Association (ASIA) scale. Both study groups underwent the same amount of cognitive training (but using a different type of training: conventional vs. innovative), consisting of three weekly sessions for eight weeks (24 sessions in total), each session lasting approximately sixty minutes, as well as the same amount of physiotherapy. The effect of the two treatments (EG/CG) was significantly different in global cognitive functioning (MOCA: *p* = 0.001), mood (BDI: *p* = 0.006), and overall quality of life (SF12 Total: *p* < 0.001), especially in physical perception (SF12-Physics: *p* = 0.004). Our results suggest that SCI patients could benefit from cognitive training using semi-immersive VR. Indeed, the integration of cognitive exercises that require movement and provide increased feedback could allow for better motor and cognitive recovery in people with SCI.

## 1. Introduction

A spinal cord injury (SCI) is damage to any part of the spinal cord, caused by a traumatic (i.e., car/motorcycle incidents, surgical complications, military wounds, gunshot) or non-traumatic (i.e., tumors, degenerative pathologies of the spine, ischemic/hemorrhagic damage) etiology [1]. Commonly, SCI is associated with a complete or partial loss of motor and sensory functions below the injury site, but it usually involves other systems as well (i.e., respiratory, cardiovascular, gastrointestinal, genito-urinary, endocrine, immunologic); thus, it can be defined as a “multisystem failure” [2]. In this context, SCI functional classification systems, such as the American Spinal Injury Association (ASIA) scale, are used to establish the severity of SCI according to the site and extent of injury [3]. In detail, the ASIA scale arranges SCI patients into five levels, from A to E, as follows: (A) a complete injury with a total loss of motor and sensory function; (B) an incomplete injury with preserved sensory function, but the motor function is lost; (C) an incomplete injury with preserved partial motor functions below the level of injury (less than half of muscles have a score of 3 in the Medical Research Council (MRC) evaluation of strength); (D) the same as C, but half of the muscles present a score of 3 in the MRC evaluation of strength; (E) normal motor and sensory examination [4]. 

From a rehabilitation point of view, conventional physiotherapy (i.e., exercise for strengthening muscles, static and dynamic balance activities, and gait training) is strongly recommended for people with SCI in order to prevent complications of being bedridden, such as muscle atrophy, pressure ulcers, and deterioration of the autonomic nervous system [5,6]. However, recent technological advances in neurorehabilitation, such as robotic devices and virtual reality (VR) systems, have been adopted to allow more intensive, repetitive, and task-oriented training, in order to achieve better outcomes [7]. Specifically, VR systems create virtual environments simulating everyday life scenarios in which patients are able to interact with different degrees of virtual immersion (i.e., non-immersive, semi-immersive, and immersive) [8]. According to a recent systematic review [9], the use of VR in the context of motor training in SCI patients can be considered a promising approach to improve sensorimotor functions, motivation, and engagement, but its effects on cognition are still unclear. 

Among the non-motor symptoms, patients with SCI may exhibit cognitive impairments in several domains, such as abstract reasoning, memory, attention, concentration, and problem solving. However, the literature on cognitive training for individuals with SCI is poor [10]. Only one previous case report [11] has suggested that cognitive training with a non-immersive VR system in a patient with traumatic SCI was useful in improving both motor and cognitive outcomes. Indeed, following SCI, individuals may experience a gap between feedback and sensory input, so rehabilitation treatment should focus on providing congruent and physiological feedback [12,13]. Hence, VR could be useful to promote multi-sensory stimulation involving the cortical sensory-motor network and other subcortical brain regions, allowing the achievement of better outcomes in motor and cognitive functions [14]. 

According to this premise, the aim of our study is to evaluate the effect of semi-immersive virtual reality cognitive training (using the BTS Nirvana, Italy) to promote global functional recovery in patients with SCI.

## 2. Materials and Methods

Patients diagnosed with SCI who attended the Neurorehabilitation Robotic Unit of the IRCCS Centro Neurolesi Bonino-Pulejo (Messina, Italy) between October 2018 and February 2020 were evaluated for inclusion in the analysis using electronic recovery data. This retrospective case-control study was conducted in accordance with the 1964 Declaration of Helsinki and approved by our Research Institute Ethics Committee (ID: IRCCS-ME-CE-19/2023). The retrospective nature of the study and extraction from an electronic medical record minimized scoring bias. The evaluations (which we collected retrospectively) were carried out at the beginning and at the end of the training by the rehabilitation team (neurologist, physiatrist, nurse, physiotherapist, and psychologist). We selected patients who had followed and completed the rehabilitation program with cognitive and motor therapy and met the inclusion criteria. The inclusion criteria were as follows: (i) age ≥ 18 years; (ii) diagnosis of SCI according to the AIS classification [15]; (iii) a stable SCI condition (i.e., at least three months after the injury); and (iv) absence of severe cognitive impairment (MoCA > 20). Exclusion criteria were: (i) presence of disabling sensory alterations, including severe visual and hearing loss; (ii) active epilepsy with frequent seizures; (iii) concomitant medical and psychiatric illness that could potentially interfere with the VR screen. 

### 2.1. Data Collection

Demographic and clinical information was collected from all included patients. The rehabilitation sessions and the evolution of the patient were recorded. Data were collected retrospectively and then analyzed. All study subjects provided general informed consent on the use of the data for research purposes, as per our research institute’s procedure.

### 2.2. Procedures

The included patients were divided into two groups with the same demographic and medical characteristics: the control group (CG), with patients who received traditional cognitive training, and the experimental group (EG), who submitted to VR training. Each group was composed of patients labeled as A or B according to the American Spinal Injury Association (ASIA) scale. A more detailed description of the two groups is included in Table 1.

All patients underwent a standard physical treatment, characterized by 10 min of warm-up with passive and active-assisted range of motion exercises for both upper and lower limbs; 10 min of strengthening exercises (i.e., isometric and isotonic muscle contractions) for lower and upper limbs; 10 min of breathing control exercises and assisted coughing techniques; 20 min of exercises related to trunk control (i.e., static and dynamic balance training, strengthening of core muscles); and 10 min of gait training.

Both study groups received the same amount of cognitive training (but different types: traditional vs. innovative), consisting of three weekly sessions, each lasting sixty minutes, for eight weeks, for a total of 24 sessions.

In both groups, the protocol defined by the rehabilitation team included a series of basic exercises, with increasing difficulties in the cognitive domains (memory, attention, and executive processes). The difficulty of the exercises raised according to the number of errors made by the patient and the execution time of the activity. 

The CG underwent traditional rehabilitation training, with individual “face-to-face” sessions between the patient and the therapist; the exercises were based on a pencil and paper method to stimulate specific cognitive skills. The EG received VR training, using the semi-immersive VR system “BTS-Nirvana” [16] during rehabilitation. All patients submitted to a clinical and neuropsychological evaluation at the beginning (T0) and at the end (T1) of the rehabilitation process.

### 2.3. Outcomes Measures

The neuropsychological battery included the Montreal Cognitive Assessment (MoCA), which consists of a quick global cognitive assessment, including specific sub-items such as attention, executive functions, memory, language, calculation, spatial orientation, visuo-constructional skills, and thinking. A score below 26 indicates a deficit in cognitive function.

The Beck Depression Inventory (BDI), a 21-item self-report outcome measure, was used to evaluate depressive symptoms, with a score greater than 10 indicating the presence of depressive symptoms.

The Short Form-12 health status questionnaire (SF12) was used to evaluate quality of life, through twelve questions regarding specific subdomains such as general health, physical functioning, role-physical, and body pain. The higher the score, the higher the quality of life. A total score above 25 indicates a good quality of life.

### 2.4. BTS-Nirvana Tool

**Figure 1:**
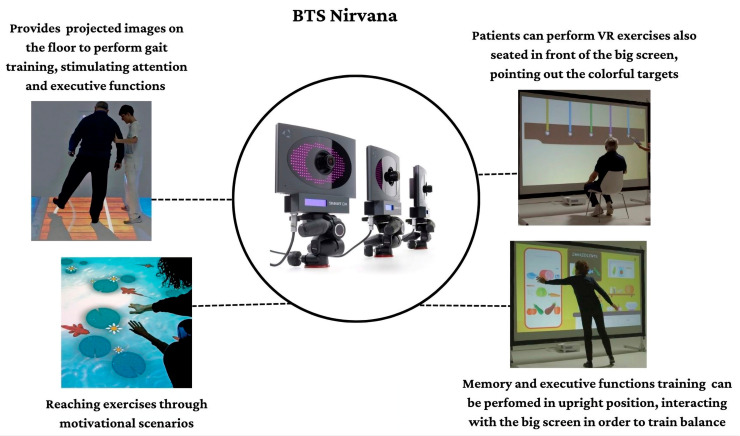
Some semi-immersive VR scenarios and modalities in which patients performed cognitive tasks joining motor skills.

The BTS-Nirvana (BTS Bioengineering, Milano, Italy) consists of computer software, two optoelectronic infrared sensors without markers, a video camera, and a projector connected to a large screen that allows patient interaction with the projected environment. In particular, patients can fully interact with the virtual environment simply through their movements, which are captured by optoelectronic infrared sensors. Some exercises (as reported in Figure 1) require the patient to perform specific activities of reaching, touching, or grabbing a series of objects, or playing with projected images (i.e., balls) on the floor, providing dual-task activities (both motor and cognitive).

This semi-immersive VR system allows the creation of a “sensory room” where the patient is involved in various realistic environments that are projected on walls and floors (see Figure 1).

The virtual environment provides strong rehabilitative audio/visual feedback through exercises, because of sounds and visual stimuli that appear when the patients succeed in hitting the target (otherwise, the object disappears). In addition, all the exercises can be personalized in real time in terms of difficulty level, execution speed, and training area [16,17]. During the VR training session, patients are free to move without obstructions and using their aids (i.e., wheelchairs, walkers, or crutches). 

### 2.5. Statistical Analysis

The medical records of 180 SCI patients who had been treated in our Spinal Cord Rehab Unit were examined. To reduce the selection bias, they were matched for age, sex, and education. The final sample consisted of 42 patients. They were assigned to the EG (21) or the CG (20). Data were entered and analyzed using the Statistics Package for Social Sciences (SPSS), version 16.0 (IBM Corp., Armonk, NY, USA). The significance level of the statistical tests was established with *p* < 0.005. Descriptive statistics were analyzed and expressed as mean ± standard deviation or as median ± first-third quartile for continuous variables, as appropriate; frequencies (%) were used for categorical variables. Nonparametric statistical tools were used to analyze the data as the Kolmogorov-Smirnov results indicated that the target variables were not normally distributed. Therefore, we used the Wilcoxon and Mann-Whitney tests for within-group and between-group comparisons, respectively, corrected for multiple comparisons (Table 2).

## 3. Results

No significant differences in age (*p* = 0.99), gender (*p* = 0.99), or education (*p* = 0.23) were found between the EG and CG (Table 1). The post-injury time interval was from four to nine months. There were no significant differences in clinical assessment scores between groups at baseline. We compared the results of the two groups at T1, observing that they differed in the scores obtained in the two treatments, with statistically significant differences in global cognitive functioning (MOCA: *p* = 0.001); mood (BDI: *p* = 0.001); and overall quality of life (SF12 Total: *p* < 0.001), especially in physical perception (SF12-Physics: *p* = 0.004). Indeed, statistical analysis showed that the experimental treatment in all patients with ASIA B led to an improvement in all test scores, except mood (BDI). On the contrary, in the EG-A (i.e., patients with ASIA A), we observed a significant improvement in global cognitive functions; overall quality of life, in terms of both mental and physical perception; and mood. Finally, in the CG-B group, we found a significant change from T0 to T1 only in mood (BDI: *p* < 0.001) (see Table 2).

## 4. Discussion

To the best of our knowledge, this is the first study that has investigated the effects of cognitive training, using a semi-immersive VR system, in SCI patients. Our results suggested that patients undergoing VR training sessions showed improvements in both cognitive functioning (MoCA) and mood (BDI) outcomes, as well as quality of life and perceived physical functioning (SF-12). Specifically, we observed that EG for both SCI-A and SCI-B obtained an increase in cognitive domains (especially in attention and visuospatial memory, as per MOCA subitems), an aspect that was not present in the GC. It has already been demonstrated that patients with SCI can develop cognitive dysfunctions in multiple domains, such as memory, attention, concentration, and executive functions [18]. These cognitive alterations may be present in the early stages of SCI, and they tend to become worse in chronic phases. This may be due to prolonged neuroinflammation processes, which determine the release of reactive oxygen species, proinflammatory cytokines, and other inflammatory molecules involving the bloodstream-to-brain areas [19]. All these mechanisms greatly increase the risk of cognitive decline, reducing the patient’s quality of life. In this vein, VR systems, through their multisensory and task-oriented approach, can promote global functional recovery, acting on use-dependent neural plasticity. As confirmed by other authors [17,18,19,20,21,22,23,24,25,26,27], the advantage of introducing cognitive training with semi-immersive VR devices is that it allows patients to be involved in the virtual environment while remaining connected to their physical surroundings at the same time. According to Leemhuis et al. [20], VR devices can also be used in SCI patients to induce some changes in the subjective experience of embodiment, due to the integration of all sensory afferents, memory, and overall cognitive functions. In fact, studies carried out using semi-immersive VR have suggested that VR training allows the integration of perception, cognition, and action, which favors the recall of memorized motor plans [21]. This effect of VR is due to the possibility of implementing realistic and highly engaging exercises (such as moving objects or grasping them), which promote an increase in control of the various sensorimotor, social, and cognitive areas [21,22,23]. 

Another possible cause of the cognitive decline is the reduction/absence of the fundamental brain sensory stimulation and the impairment of the sensory-motor pathway. In SCI, sensorimotor signals between the brain and the body parts below the level of the lesion are disrupted, although the physical body remains unaffected [24,25,26,27]. This causes motor, sensory, and, as observed in our sample, also cognitive problems that are often overlooked in rehabilitation. As a result, VR-based rehabilitation treatment may provide coherent physiological feedback to individuals with SCI who may have de-afferents producing discrepancies in feedback and sensory information [26,27]. Indeed, feedback becomes fundamental in neurorehabilitation practice since it enables the user to adjust their movement or intention in the most appropriate direction, according to the virtual environment. In fact, the immediate feedback provided by VR teaches the body and brain how to correct patients’ movements, according to what they have learned (reinforcement learning). Studies on multiple sclerosis patients have emphasized that VR combines cognitive exercises with imitation components and motor sequences, whereby the patient performs movements in the correct sequence, providing increased visual and auditory inputs, with double reinforcement that involves both motor and cognitive functions [28,29]. To support this issue, Scandola et al. investigated the representation of peripersonal space using VR and demonstrated that SCI patients reported motor benefits when they were exposed to visual-motor feedback [30]. Thus, the intense, repetitive, task-oriented practice provided by VR may have a major impact on cortical neural plasticity thanks to multisensory stimulation. In particular, VR feedback can exert an input from higher-level networks to the basic ones, acting on motor programming and influencing visuomotor and sensorimotor areas and peripheral structures [31]. The BTS-Nirvana, through semi-immersive VR, could have allowed the integration of perception, cognition, and action in our sample, increasing, as mentioned, the recovery of the memorized motor plans through “reinforcement learning” with better cognitive performances and improving the perception of one’s motor well-being.

Concerning the improvement in depressive symptoms, we found that both CG-A and EG-B groups improved after the training, confirming that mood may be affected by the rehabilitation training. However, better scores were found following the VR training. Growing evidence is demonstrating the potential efficacy of VR in improving anxiety disorders and depression, although more research is needed, especially concerning mood disorders [32]. Although we are not completely able to state how VR can affect mood and other behavioral problems, we believe that motivation and enjoyment, as well as the better improvement in cognition and motor skills following training in a virtual environment, may play an important role. This hypothesis may also explain the significant improvement in quality of life (as per SF-36) that we found in the EG as compared to the CG. In a VR environment, patients can be trained in a more ecological way, performing those activities that are too dangerous to perform in real life; therefore, they may better potentiate their motor skills with positive results on both mood and cognition. In fact, this kind of VR-related “dual-task” training is known to lead to better outcomes in neurological patients [33], as confirmed by our study on SCI. 

This retrospective study has some limitations to acknowledge, in addition to the retrospective design itself. The sample size is small, and the results may not be generalizable to the SCI population. The absence of assessment of other variables, including changes in drug treatment, could have influenced the results. We also did not evaluate data on motor functioning. However, this study should be considered as an exploratory study, which needs further clinical studies on larger samples, with more specific motor and cognitive outcome measures, to confirm our promising results.

## 5. Conclusions

In conclusion, our results suggest that SCI patients could benefit from cognitive training implemented through the use of semi-immersive VR. Indeed, the integration of cognitive exercises that require movement and provide increased feedback could allow cognitive and global physical functioning recovery in people with SCI. Further studies with larger sample sizes, using neuroimaging and electrophysiological tools, are needed to confirm these promising findings and to better understand the neural basis of cognitive dysfunction and recovery following SCI.

## Figures and Tables

**Table 1:** Demographic and clinical characteristics of the patients.

	Experimental Group	Control Group	All	*p*-Value
Patients	21	21	42	
Age (years)	58.6 ± 15.0	58.6 ± 9.0	58.6 ± 12.6	0.99
Gender				0.99
Female	10 (40.0%)	10 (40.0%)	20 (40.0%)	
Male	11 (60.0%)	11 (60.0%)	22 (60.0%)	
Education				0.23
Elementary school	-	-	-	
Middle school	5 (23.8%)	3 (14.3%)	8 (19.0%)	
High school	14 (66.7%)	12 (57.1%)	26 (62.0%)	
University	2 (9.5%)	6 (28.6%)	8 (19.0%)	
Spinal Injury Disability (ASIA)				0.99
ASIA—A patients	10 (47.6%)	10 (47.6%)	20 (47.6%)	
ASIA—B patients	11 (52.3%)	11 (52.3%)	22 (52.3%)	
Time Post—Injury				0.93
AIS—A patients	7 ± 1	6 ± 2	7 ± 2	
AIS—B patients	6 ± 2	7 ± 2	7 ± 2	

Mean ± standard deviations were used to describe continuous variables; proportions (numbers and percentages) were used to describe categorical variables.

**Table 2:** Statistical comparison of changes in the clinical score of patients from T0 to T1.

Clinical Scale		Mean (DS)	*p*-Value
MOCA	CG-A T0/EG-A T0	21.4 (4.3)–22.2 (2.3)	0.60
	CG-B T0/EG-B T0	21.3 (4.1)–23.4 (2.2)	0.15
	CG-A T1/EG-A T1	22.7 (4.3)–25.8 (1.9)	0.05
	CG-B T1/EG-B T1	21.6 (3.5)–26.1 (1.9)	**<0.001**
	CG-A T0/CG-A T1	21.4 (4.3)–22.7 (4.3)	0.006
	CG-B T0/CG-B T1	21.3 (4.1)–21.6 (3.5)	0.50
	EG-A T0/EG-A T1	22.2 (2.3)–25.8 (1.9)	**<0.001**
	EG B T0-EG B T1	23.4 (2.2)–26.1 (1.9)	**<0.001**
	CG A T0-CG B T0	21.4 (4.3)–21.3 (4.1)	0.94
	CG A T1-CG B T1	22.7 (4.3)–22.6 (3.5)	0.54
	EG A T0-EG B T0	22.2 (2.3)–23.4 (2.2)	0.25
	EG A T1-EG B T1	25.8 (1.9)–26.1 (1.9)	0.74
	CG A T0-EG A T1	21.4 (4.3)–25.8 (1.9)	0.008
	EG B T0-CG B T1	23.4 (2.2)–21.6 (3.5)	0.18
	EG B T1-CG B T0	26.1 (1.9)–21.3 (4.1)	**<0.001**
BDI	CG-A T0/EG-A T0	17.5 (6.3)–12.5 (7.3)	0.12
	CG-B T0/EG-B T0	14.5 (6.1)–107 (7.6)	0.21
	CG-A T1/EG-A T1	16.6 (4.3)–5.6 (6.3)	**<0.001**
	CG-B T1/EG-B T1	12.5 (6.2)–5.2 (4.8)	0.009
	CG-A T0/CG-A T1	17.5 (6.3)–16.6 (14.3)	0.43
	CG-B T0/CG-B T1	14.5 (6.1)–12.5 (6.2)	**<0.001**
	EG-A T0/EG-A T1	12.5 (7.3)–5.6 (6.3)	**<0.001**
	EG B T0-EG B T1	10.7 (7.6)–5.2 (4.8)	0.14
	CG A T0-CG B T0	17.5 (6.3)–14.5 (6.1)	0.29
	CG A T1-CG B T1	16.6 (4.3)–12.5 (6.2)	0.09
	EG A T0-EG B T0	15.5 (7.3)–10.7 (7.6)	0.59
	EG A T1-EG B T1	5.6 (6.3)–5.2 (4.8)	0.87
	CG A T0-EG A T1	17.50 (6.3)–12.5 (7.3)	**<0.001**
	EG B T0-CG B T1	10.7 (7.6)–12.5 (6.2)	0.54
	EG B T1-CG B T0	5.2 (4.8)–14.5 (6.1)	**<0.001**
SF-12 TOTAL	CG-A T0/EG-A T0	27.8 (8.1)–21.1 (5.7)	0.39
	CG-B T0/EG-B T0	24.5 (8.2)–26.9 (6.2)	0.45
	CG-A T1/EG-A T1	30.3 (10.2)–35 (4.5)	0.19
	CG-B T1/EG-B T1	26.1 (7.5)–35.7 (6.4)	**<0.001**
	CG-A T0/CG-A T1	27.8 (8.1)–30.3 (10.2)	0.19
	CG-B T0/CG-B T1	24.5 (8.2)–26.1 (7.5)	0.03
	EG-A T0/EG-A T1	21.1 (5.7)–35 (4.5)	**<0.001**
	EG B T0-EG B T1	26.9 (6.2)–35.7 (6.4)	**<0.001**
	CG A T0-CG B T0	27.8 (8.1)–24.5 (8.2)	0.37
	CG A T1-CG B T1	30.3 (10.2)–26.1 (7.5)	0.29
	EG A T0-EG B T0	21.1 (5.7)–26.9 (6.2)	0.49
	EG A T1-EG B T1	35 (4.5)–35.7 (6.4)	0.77
	CG A T0-EG A T1	27.8 (8.1)–35 (4.5)	0.02
	EG B T0-CG B T1	26.9 (6.2)–26.1 (7.5)	0.78
	EG B T1-CG B T0	35.7 (6.4)–24.5 (8.2)	**<0.001**
SF-12 PHYSICAL	CG-A T0/EG-A T0	15.4 (5.3)–12.2 (2.4)	0.01
	CG-B T0/EG-B T0	13.3 (3.6)–11.9 (3.5)	0.38
	CG-A T1/EG-A T1	15.2 (4.7)–16.8 (2.6)	0.36
	CG-B T1/EG-B T1	15 (3.7)–16.5 (2.4)	0.29
	CG-A T0/CG-A T1	15.4 (5.3)–15.2 (4.7)	0.90
	CG-B T0/CG-B T1	13.3 (3.6)–15 (3.7)	0.07
	EG-A T0/EG-A T1	12.2 (2.4)–16.8 (2.6)	**<0.001**
	EG B T0-EG B T1	11.9 (3.5)–16.5 (2.4)	**<0.001**
	CG A T0-CG B T0	15.4 (5.3)–13.3 (3.6)	0.29
	CG A T1-CG B T1	15.2 (4.7)–15 (3.7)	0.91
	EG A T0-EG B T0	12.2 (2.4)–11.9 (3.5)	0.83
	EG A T1-EG B T1	16.8 (2.6)–16.5 (2.4)	0.75
	CG A T0-EG A T1	15.4 (5.3)–16.8 (2.6)	0.46
	EG B T0-CG B T1	11.9 (3.5)–15 (3.7)	0.06
	EG B T1-CG B T0	16.5 (2.4)–13.3 (3.6)	0.02

Significant differences are in bold. Legend: EG = experimental group; CG = control group; T0 = evaluation at baseline; A = ASIA A; B=ASIA B; T1 = evaluation at the end of the protocol; MOCA = Montreal Cognitive Assessment; BDI = Beck Depression Inventory; SF-12 TOTAL = Short Form-12 Health Survey Total; SF-12 PHYSICAL = Short Form-12 Health Survey Physical.

## Data Availability

Data will be available on request to the corresponding author.
